# A Population-Based Cohort Study of *Mycobacterium tuberculosis* Beijing Strains: An Emerging Public Health Threat in an Immigrant-Receiving Country**?**

**DOI:** 10.1371/journal.pone.0038431

**Published:** 2012-06-05

**Authors:** Deanne Langlois-Klassen, Dennis Kunimoto, L. Duncan Saunders, Linda Chui, Jody Boffa, Dick Menzies, Richard Long

**Affiliations:** 1 Department of Public Health Sciences, University of Alberta, Edmonton, Canada; 2 Tuberculosis Program Evaluation and Research Unit, University of Alberta, Edmonton, Canada; 3 Department of Medicine, University of Alberta, Edmonton, Canada; 4 Department of Laboratory Medicine and Pathology, University of Alberta, Edmonton, Canada; 5 Provincial Laboratory for Public Health, Edmonton, Canada; 6 Respiratory Division, McGill University Health Centre and McGill University, Montreal, Canada; St. Petersburg Pasteur Institute, Russian Federation

## Abstract

**Introduction:**

*Mycobacterium tuberculosis* Beijing strains are frequently associated with tuberculosis outbreaks and drug resistance. However, contradictory evidence and limited study generalizability make it difficult to foresee if the emergence of Beijing strains in high-income immigrant-receiving countries poses an increased public health threat. The purpose of this study was to determine if Beijing strains are associated with high risk disease presentations relative to other strains within Canada.

**Methods:**

This was a retrospective population-based study of culture-confirmed active TB cases in a major immigrant-receiving province of Canada in 1991 through 2007. Of 1,852 eligible cases, 1,826 (99%) were successfully genotyped. Demographic, clinical, and mycobacteriologic surveillance data were combined with molecular diagnostic data. The main outcome measures were site of disease, lung cavitation, sputum smear positivity, bacillary load, and first-line antituberculosis drug resistance.

**Results:**

A total of 350 (19%) patients had Beijing strains; 298 (85%) of these were born in the Western Pacific. Compared to non-Beijing strains, Beijing strains were significantly more likely to be associated with polyresistance (aOR 1.8; 95% CI 1.0–3.3; p = 0.046) and multidrug-resistance (aOR 3.4; 1.0–11.3; p = 0.049). Conversely, Beijing strains were no more likely than non-Beijing strains to be associated with respiratory disease (aOR 1.3; 1.0–1.8; p = 0.053), high bacillary load (aOR 1.2; 0.6–2.7), lung cavitation (aOR 1.0; 0.7–1.5), immediately life-threatening forms of tuberculosis (aOR 0.8; 0.5–1.6), and monoresistance (aOR 0.9; 0.6–1.3). In subgroup analyses, Beijing strains only had a significant association with multidrug-resistant tuberculosis (aOR 6.1; 1.2–30.4), and an association of borderline significance with polyresistant tuberculosis (aOR 1.8; 1.0–3.5; p = 0.062), among individuals born in the Western Pacific.

**Conclusion:**

Other than an increased risk of polyresistant or multidrug-resistant tuberculosis, Beijing strains appear to pose no more of a public health threat than non-Beijing strains within a high-income immigrant-receiving country.

## Introduction

Since first being reported in 1995 (Beijing isolates) and 1996 (strain W) [1], [2], the Beijing lineage of *Mycobacterium tuberculosis* (also referred to as the East Asian lineage or Lineage 2) has garnered much attention in international tuberculosis literature. The largest genotype family of *M. tuberculosis* [3], Beijing strains account for 13% of strains globally and dominate the *M. tuberculosis* epidemiology in some geographic areas [4], [5]. In the Western Pacific countries of China, Japan, South Korea and Vietnam, 54–92% of *M. tuberculosis* case isolates are Beijing strains [1], [6]–[10]. While other countries are only now experiencing an emergence of Beijing lineage strains [4], [11]–[15], China has had high endemic levels of these strains for at least 60 years [16].

Active tuberculosis disease (TB) resulting from infection with Beijing strains has frequently been associated with TB outbreaks [2], [11], antituberculosis drug resistance [4], [8], [17], [18], treatment failure [19] and relapse [19]–[21]. Of particular concern is the association between Beijing strains and multidrug-resistant TB (MDR-TB) [18], [22], [23]. Beijing strains also appear to have an enhanced ability to circumvent immunity induced through bacille Calmette-Guérin (BCG) vaccination, potentially resulting in a selective advantage of these strains in populations with high rates of BCG vaccination [24]–[26].

In contrast, other studies have found no significant associations between Beijing strains and either BCG vaccination status [27] or various presentations of TB [4], [8], [23], [28]–[32]. This inter-study variability may result from the heterogeneous distribution of Beijing sublineages; programmatic differences in TB control; inherited and acquired host factors; socioeconomic circumstances; chance; and other factors [33]–[35]. Furthermore, little clarity is afforded by evidence of genotypic diversity within the *M. tuberculosis* species because it remains inconclusive as to whether or not genotypic diversity meaningfully influences the outcome of infection *in vivo* [36]. The bottom line – the epidemiologic significance of Beijing lineage strains in the human population remains largely ambiguous.

Contradictory evidence within the Beijing literature and the often limited generalizability of studies make it difficult to foresee whether the emergence of Beijing lineage strains in high-income immigrant-receiving countries with low TB incidence (hereafter referred to as immigrant-receiving countries for brevity) poses an increased public health threat. This study sought to determine if the Beijing lineage of *M. tuberculosis* strains was associated with more high risk presentations of active TB than other strains in Canada, a country with one of the highest levels of immigration per capita internationally and in which a quarter of the foreign-born population has originated from the Western Pacific [37], [38]. A secondary objective was to determine if Beijing disease presentation varied in relation to patients’ age or population group.

## Methods

### Ethics Statement

Study approval was obtained from the University of Alberta Health Research Ethics Board. The need for patient’s informed consent was waived by the University of Alberta Health Research Ethics Board as the retrospective analysis of anonymous and routine surveillance data did not require direct patient contact.

### Study Population

This retrospective cohort study investigated cases of active TB diagnosed among residents of the immigrant-receiving province of Alberta, Canada (population of 3,290,355 in 2006) from January 1, 1991 through June 30, 2007. Canadian-born individuals were born in Canada or born in a foreign country to Canadian parents; all others were foreign-born. Aboriginals (defined in this study as First Nations peoples registered with Indian and Northern Affairs Canada) were distinguished from Canadian-born ‘other’ (non-Status Indian, Métis, Inuit, and non-Aboriginal individuals) due to a marked disparity in the TB rates of these groups. Foreign-born individuals were grouped into those born in the Western Pacific region (Table S1) [39] and those born elsewhere (foreign-born ‘other’) given the high prevalence of Beijing strains in the Western Pacific.

Foreign- and Canadian-born population estimates were obtained from Canadian censuses (1991, 1996, 2001 and 2006) using customized reports from Statistics Canada. To calculate person-years, these census estimates were combined with estimates between census years as calculated with linear interpolation as well as the population estimates for 2007 which were obtained through linear extrapolation. Estimates for Canadian-born ‘other’ were those derived from the censuses minus annual Aboriginal population estimates as obtained directly from Indian and Northern Affairs Canada.

### Cases

All culture-confirmed active TB cases diagnosed during the study period as per the Alberta Tuberculosis Registry were eligible for study inclusion. Demographic and clinical data from the TB registry were combined with data from the Provincial Laboratory for Public Health (‘Provincial Laboratory’) where all of the mycobacteriology in the province is performed.

Cases were grouped by site (respiratory versus non-respiratory disease) and severity (immediately life-threatening forms of TB versus other forms of TB). Respiratory cases consisted of: primary, pleural, pulmonary or ‘other respiratory’ TB (ICD-9 codes 010–012) [40]; miliary TB (ICD-9 code 018) with culture-positive respiratory specimen(s); and cases with concurrent respiratory and non-respiratory TB. Miliary TB and TB involving the central nervous system (CNS) (ICD-9 code 013) comprised immediately life-threatening forms of TB.

The infectiousness of respiratory cases was evaluated in relation to sputum smear positivity and the presence of lung cavitation on chest radiograph. Semi-quantitative scores for acid-fast bacilli (AFB) load on baseline sputum smears were also analyzed for respiratory cases diagnosed after 1992 that had positive sputum smears collected on or before the date of diagnosis (the start date of treatment); this data was unavailable for cases in 1991–1992.

Monoresistant-TB refers to resistance to a single first-line antituberculosis drug, namely isoniazid (INH), rifampin (RMP), pyrazinamide (PZA), ethambutol (EMB), or streptomycin (STM). Polyresistance was defined as resistance to two or more first-line antituberculosis drugs but not to both INH and RMP; resistance to at least INH and RMP constituted MDR-TB. ‘Any first-line drug resistance’ includes monoresistant-TB, polyresistant-TB, and MDR-TB.

Cases were also dichotomized as being a new active case (first episode of TB) or retreatment TB case (history of a previous episode of TB).

### Laboratory Methods

The Provincial Laboratory conducted all routine mycobacteriologic studies as per the Canadian Tuberculosis Standards [40] and completed routine DNA fingerprinting as previously described using IS*6110* restriction fragment length polymorphism (RFLP) typing and, for isolates with five copies or less of IS*6110*, spoligotyping [41]. Clusters were defined as groups of two or more patients with identical RFLP patterns and, for isolates with five or fewer copies of IS*6110,* identical spoligotype patterns.

Isolates were assigned to an *M. tuberculosis* lineage according to the PCR-based detection of large sequence polymorphisms (LSPs) [31], [42], [43]. The Provincial Laboratory analyzed LSPs with an ABI 7000 Real-Time PCR machine (Azco Biotech, Inc., San Diego, CA) using standard conditions and published TaqMan™ primers and probes [42], [43]. Isolates with a deletion of RD105 were categorized as Beijing lineage strains and all others as non-Beijing lineage strains (hereafter referred to as Beijing strains and non-Beijing strains). Accordingly, this methodology assigned both classical Beijing strains (those with concurrent deletions of RD105 and RD207, the latter resulting in the characteristic ‘Beijing’ spoligotype pattern consisting of a loss of spacers 1 to 34 and a presence of ≥3 spacers among spacers 35–43) [3] and ancestral members of this family (those with a deletion of RD105 but without a concurrent deletion of RD207) to the same lineage [44].

The lineage assignment of a convenience sample of isolates was confirmed through spoligotyping at the Provincial Laboratory or through LSP analyses at extra-provincial laboratories (M. Behr, McGill University; C. Pepperell, Stanford University).

### Statistical Analysis

Agreement in the lineage assignment of isolates between the initial LSP analysis and confirmatory testing was assessed with the Kappa co-efficient. The incidence rate ratio (RR) was used to compare TB rates between Beijing and non-Beijing strains overall as well as between groups defined on the basis of sex, age at diagnosis, and population group within each lineage. Associations between lineage and various demographic and disease variables were evaluated with binary or multinomial logistic regression; p-values correspond to the likelihood ratio chi-square test in bivariate models [45]. Sex, age at diagnosis, and population group frequently confounded the associations between *M. tuberculosis* lineage and disease presentation (>15% change in the estimated coefficient) and were therefore included in multivariate analyses [46]. Additional adjustment was completed to ensure that associations with disease presentation were independent of HIV co-infection and associations with drug resistance were not confounded by previous TB or clustering. Evidence for effect modification within multivariate analyses was based on the likelihood ratio test [45]. Subgroup analysis was planned *a priori* to enhance the transparency of potential differences in Beijing disease presentation across population group (Western Pacific versus others) and age strata [46]. All statistical tests used a 5% level of significance and 95% confidence intervals (CI) were calculated where appropriate; p-values that were p>0.05 but p<0.07 were considered to be of borderline statistical significance. Statistical analyses were conducted with Stata/IC 11 (StataCorp. 2009. *Stata Statistical Software: Release 11.* College Station, TX: StataCorp LP).

## Results

The Alberta TB Registry was notified of 1852 culture-confirmed *M. tuberculosis* cases from 1991–2007 and isolates from 1827 (99%) cases were available for LSP analysis. Confirmatory genotyping was completed on a sample of 535 (29%) cases using spoligotyping (n = 412), LSP analysis at an external laboratory (n = 98) or both spoligotyping and LSP analysis (n = 25). One case was subsequently removed from the study due to discordance in lineage assignment based on initial and confirmatory LSP analyses (K = 0.995; p<0.001). The demographic attributes of included and excluded cases were similar (Table S2). The convenience sample included 119 (22%) cases with Beijing lineage strains and 110 (92%) of these were spoligotyped. All of these Beijing lineage isolates were found to have characteristic Beijing spoligotype patterns [3] and no “pseudo-Beijing” isolates (described as isolates with an intact RD105 but with a characteristic Beijing spoligotype pattern) [47] were identified.

Of the 1826 cases included in the study, 350 (19%) were Beijing strains and the associated incidence rate was 0.2 (95% CI 0.2–0.3) times that of other strains (rates of 0.7 and 3.1 per 100,000 person-years, respectively) (Table 1). The lineages also had marked differences in the distribution of cases by age (p = 0.004) and population group (p<0.0001) but not sex (p = 0.398).

**Table 1:** Incidence of Beijing and Non-Beijing strains of *M. tuberculosis* in Alberta, 1991–2007.

		Beijing Strains			Non-Beijing Strains		
Characteristic	PYRs*	No. (%)	Rate†	RR (95%CI)	No. (%)	Rate†	RR (95%CI)
Sex							
Female	235.4	161 (46.0)	0.7	1.0	716 (48.5)	3.0	1.0
Male	235.7	189 (54.0)	0.8	1.2 (0.9, 1.5)	760 (51.5)	3.2	1.1 (1.0, 1.2)
Age at Diagnosis							
<35 years	243.9	104 (29.7)	0.4	1.0	438 (29.7)	1.8	1.0
35–64 years	180.9	109 (31.1)	0.6	1.4 (1.1, 1.9)	583 (39.5)	3.2	1.8 (1.6, 2.0)
>64 years	46.3	137 (39.1)	3.0	6.9 (5.3, 9.1)	455 (30.8)	9.8	5.5 (4.8, 6.3)
Population Group							
CB Other	381.4	15 (4.3)	0.04	1.0	324 (22.0)	0.8	1.0
CB Aboriginal	13.6	5 (1.4)	0.4	9.4 (2.7, 27.1)	288 (19.5)	21.2	25.0 (21.2, 29.4)
FB Other	56.5	32 (9.1)	0.6	14.6 (7.7, 29.0)	469 (31.8)	8.4	9.9 (8.6, 11.4)
FB Western Pacific	19.6	298 (85.1)	14.6	371.7 (221.7, 672.5)	395 (26.8)	19.4	22.8 (19.6, 26.5)
Total	471.1	350 (100.0)	0.7		1476 (100.0)	3.1	

Abbreviations: PYRs, person-years of observation; RR, incidence rate ratio; CI, confidence interval; CB, Canadian-born; FB, foreign-born; WP, Western Pacific.

* Estimates of the foreign-born and Canadian-born populations were derived from customized Statistics Canada census reports. Estimates for Canadian-born ‘other’ were those derived from the censuses minus the annual population of Canadian-born Aboriginal peoples as obtained directly from Indian and Northern Affairs Canada.

† Crude incidence rate per 100,000 person-years.

Overall, 3% of Canadian-born patients and 28% of foreign-born patients had Beijing strains. Foreign-born individuals accounted for 330 (94%) Beijing cases and 298 (90%) of these were born in the Western Pacific (Table 1). More specifically, 282 (81%) Beijing cases were born in the Western Pacific’s Beijing ‘hotspots’ of China (including Hong Kong, Macau, and Taiwan), Japan, South Korea, and Vietnam. Consequently, the incidence rate of Beijing strains among individuals born in the ‘hotspots’ was nine (95% CI 5.6–16.5) times that of persons born elsewhere in the Western Pacific, 37 (95% CI 25.5–54.8) times that of foreign-born ‘other’, and 417 (95% CI 265.0–693.4) times that of Canadian-born persons. It is also noteworthy that only 20 Canadian-born cases had Beijing strains (five being Aboriginals) and that the proportion of Beijing strains in the Canadian-born non-Aboriginal group was significantly higher than that of Aboriginal peoples (OR 0.4; CI 0.1–1.0; p = 0.046).

**Figure 1:**
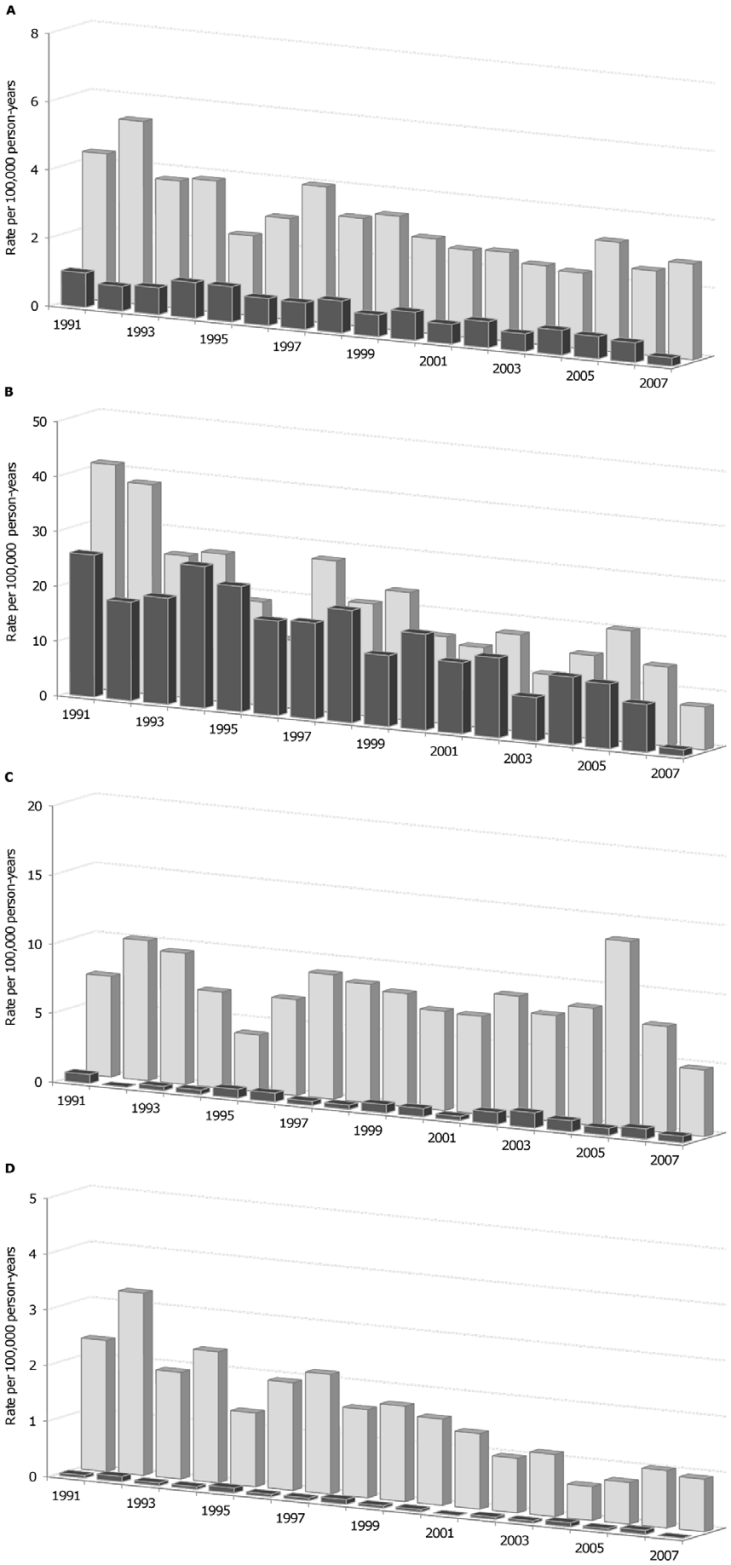
Annual incidence rates of Beijing and non-Beijing lineage strains by population group. In each panel: rates are per 100,000 person-years (y-axis); time corresponds to the year of diagnosis (x-axis); bars with dark grey shading are cases with Beijing strains; and bars with light grey shading are cases with non-Beijing strains. The panels represent total cases (panel A) as well as cases within the foreign-born Western Pacific (panel B), foreign-born ‘other’ (panel C) and Canadian-born (panel D) populations.

There was an overall trend of declining annual incidence rates of Beijing strains (Figure 1A). This primarily reflected the reduction in Beijing incidence rates in the foreign-born Western Pacific (Figure 1B) given the trend of slightly increasing Beijing rates in the foreign-born ‘other’ (Figure 1C) and the inconsequential rates of Beijing strains in the Canadian-born (Figure 1D). Apart from a pronounced reduction in the incidence rates of non-Beijing strains in the Canadian-born (Figure 1D), the trends in the annual incidence rates of non-Beijing strains mimicked that of Beijing strains both overall (Figure 1A) and within each foreign-born group (Figure 1B and Figure 1C).

The DNA fingerprint patterns of *M. tuberculosis* isolates from 1821 (99.7%) patients were available of which 348 (19%) were Beijing strains and 1473 (81%) were non-Beijing strains. Sixty (17%) patients with Beijing strains were associated with 22 clusters (ranging in size from 2 to 6 patients per cluster) compared to 560 (38%) patients with non-Beijing strains in 139 clusters (2 to 43 patients per cluster), the proportion of clustered Beijing cases being significantly lower than that of non-Beijing cases (p<0.0001). Although a smaller proportion of Beijing cases were clustered relative to non-Beijing cases among patients born in the Western Pacific (15.5% versus 27.5%, p<0.0001) and those born in Canada (35.0% versus 62.0%, p = 0.015), a similar proportion of clustering was observed for Beijing and non-Beijing cases among foreign-born patients who were born outside of the Western Pacific (21.9% versus 15.6%, p = 0.346). Thirteen (59%) of the 22 Beijing clusters consisted exclusively of foreign-born Western Pacific patients and all but one of these were limited to patients born in Beijing ‘hotspot’ countries. Six of the remaining nine Beijing clusters also involved patients born in the Western Pacific ‘hotspot’ countries as well as either a Canadian-born patient (two clusters), a foreign-born ‘other’ patient (three clusters) or both a Canadian-born patient and a foreign-born ‘other’ patient (one cluster). The three exceptions were one cluster composed of three Canadian-born individuals (one Aboriginal person), one cluster composed of two individuals born in the same country within the former Union of Soviet Socialist Republics, and one cluster composed of a Canadian-born patient and a foreign-born ‘other’ (India) patient.

Respiratory TB, sputum smear positivity, high bacillary load or lung cavitation were no more likely among Beijing cases than non-Beijing cases in unadjusted analysis (Table 2). However, after controlling for the demographic variables of sex, age at diagnosis and population group, an increased likelihood of respiratory TB among Beijing cases was of borderline significance (Table 2).

**Table 2:** Association between *M. tuberculosis* lineage and disease presentation in Alberta, 1991 to mid-2007.

	Beijing (n = 350)	Non-Beijing (n = 1476)		
Disease Presentation	No. (%)	No. (%)	OR (95% CI)*	aOR (95% CI)†
Respiratory TB	259 (74.0)	1122 (76.0)	0.9 (0.7, 1.2)	1.3 (1.0, 1.8)**
Sputum smear positive‡	112 (45.9)	538 (50.9)	0.8 (0.6, 1.1)	1.1 (0.8, 1.5)
High bacillary load§	16 (29.6)	89(33.3)	0.8 (0.4, 1.6)	1.2 (0.6, 2.7)
Lung cavitation	52 (14.9)	268 (18.2)	0.8 (0.6, 1.1)	1.0 (0.7, 1.5)
Immediately life-threatening TB	16 (4.6)	99 (6.7)	0.7 (0.4, 1.1)	0.8 (0.5, 1.6)
Any first-line drug resistance	73 (20.9)	161 (10.9)	2.2 (1.6, 2.9)	1.2 (0.8, 1.6)
Monoresistance	41 (11.7)	120 (8.1)	1.6 (1.1, 2.4)	0.9 (0.6, 1.3)
INH	11 (3.1)	48 (3.3)	1.0 (0.5, 2.0)	0.4 (0.2, 0.9)
RMP	0	0	–	–
PZA	1 (0.3)	8 (0.6)	0.4 (0.2, 0.6)	0.7 (0.4, 1.2)
EMB	0	2 (0.1)	–	–
STM	29 (8.3)	62 (4.2)	2.2 (1.4, 3.5)	1.4 (0.8, 2.3)
Polyresistance	25 (7.1)	34 (2.3)	3.5 (2.0, 5.9)	1.8 (1.0, 3.3)††
MDR-TB	7 (2.0)	7 (0.5)	4.7 (1.7, 13.6)	3.4 (1.0, 11.3)‡‡

Abbreviations: OR, odds ratio; aOR, adjusted odds ratio; CI, confidence interval; INH, isoniazid; RMP, rifampin; PZA, pyrazinamide; EMB, ethambutol; STM, streptomycin; MDR-TB, multidrug-resistant TB.

* Non-Beijing strains are the reference group; –, logistic regression could not be completed due to cell count(s) of zero.

† Association between Beijing lineage and each disease presentation after adjusting for sex, age, and population group; non-Beijing strains are the reference group.

‡ There were 1301 respiratory TB cases with data related to airway secretions, 1057 (81.2%) and 244 (18.8%) being attributed to non-Beijing and Beijing lineages, respectively.

§ There were 321 respiratory TB cases diagnosed after 1992 that had sputum smear-positive specimens collected on or before the date of diagnosis.

** p = 0.053.

†† p = 0.046.

‡‡ p = 0.049.

Compared to non-Beijing cases, Beijing cases were not associated with immediately life-threatening forms of TB in unadjusted or adjusted analysis (Table 2).

All isolates were tested for INH, RMP, and EMB susceptibility and all but one isolate for STM susceptibility. As well, 1609 (88%) isolates were tested for PZA susceptibility (50% in 1991–1993 and 99% in 1994–2007). Overall, 1592 (87%) isolates were pan-sensitive to the antituberculosis drugs for which they were screened. Beijing strains were not associated with an increased likelihood of any first-line antituberculosis drug resistance or monoresistance in adjusted analysis (Table 2). Rather, polyresistance and MDR-TB were two to three times more likely in Beijing isolates than other isolates independent of demographic factors, albeit with a wider CI around the estimate for MDR-TB (Table 2). These associations between Beijing strains and drug resistance were not confounded by previous TB or clustering (data not shown). Of potential clinical importance was the finding that six (86%) of the seven Beijing isolates with MDR were also resistant to at least two other first-line drugs (EMB, PZA and/or STM) and all but one of these were diagnosed among individuals born in the Beijing ‘hotspots’.

A total of 903 (49%) TB patients had consented to HIV antibody testing. However, as all co-infected patients were aged 15–64 years when diagnosed with TB, analysis in relation to HIV status was subsequently limited to the 705 TB cases in this age group who had undergone HIV testing. Beijing strains were associated with 103 (15%) of these TB cases, five (5%) of which were HIV co-infected. Among TB cases with non-Beijing strains, 46 (8%) of 556 were co-infected with HIV. Relative to non-Beijing strains, Beijing strains were not associated with TB-HIV co-infection in unadjusted or adjusted analysis (Table S3). After adjusting for HIV status and demographic variables, there was also no association between Beijing strains and disease presentation apart from that of polyresistance (aOR 2.8; 95% CI 1.2–6.7) (Table S3).

The associations between Beijing strains and disease presentation were not consistent in all subgroups (Table 3 and Table 4). An association between Beijing strains and MDR-TB was only noted in the foreign-born Western Pacific (Table 3). Additionally, Beijing strains were associated with polyresistance in those aged <35 years at diagnosis (Table 4) whereas this association was of borderline significance among individuals born in the Western Pacific (Table 3). As well, Beijing strains were associated with sputum smear-positive respiratory TB among those aged <35 years at diagnosis but not those aged ≥35 years at diagnosis (Table 4).

**Table 3:** Association between *M. tuberculosis* lineage and disease presentation based on population group.

	Foreign-born Western Pacific			All Others*		
	Beijing	Non-Beijing		Beijing	Non-Beijing	
	(n = 298)	(n = 395)		(n = 52)	(n = 1081)	
Disease Presentation	No. (%)	No. (%)	aOR (95% CI)†	No. (%)	No. (%)	aOR (95% CI)†
Respiratory TB	214 (71.8)	256 (64.8)	1.2 (0.8, 1.7)	45 (86.5)	866 (80.1)	1.6 (0.7, 3.6)
Sputum smear-positive‡	95 (47.3)	91 (38.1)	1.5 (1.0, 2.1)§	17 (39.5)	447 (54.7)	0.6 (0.3, 1.0)**
High bacillary load††	12 (28.6)	9 (19.6)	1.7 (0.6, 4.7)	4 (33.3)	80 (36.2)	0.8 (0.2, 2.7)
Lung cavitation	43 (14.4)	54 (13.7)	1.1 (0.7, 1.7)	9 (17.3)	214 (19.8)	0.8 (0.4, 1.6)
Immediately life-threatening TB	13 (4.4)	20 (5.1)	0.8 (0.4, 1.6)	3 (5.8)	79 (7.3)	0.8 (0.2, 2.7)
Any first-line drug resistance	67 (22.5)	87 (22.0)	1.1 (0.8, 1.6)	6 (11.5)	74 (6.8)	1.6 (0.6, 3.9)
Monoresistance	37 (12.4)	66 (16.7)	0.8 (0.5, 1.3)	4 (7.7)	54 (5.0)	1.5 (0.5, 4.3)
Polyresistance	23 (7.7)	19 (4.8)	1.8 (1.0, 3.5)‡‡	2 (3.9)	15 (1.4)	2.5 (0.5, 11.3)
MDR-TB	7 (2.4)	2 (0.5)	6.1 (1.2, 30.4)	0	5 (0.5)	–

Abbreviations: aOR, adjusted odds ratio; CI, confidence interval; TB, tuberculosis; MDR-TB, multidrug-resistant tuberculosis; –unable to calculated.

* Individuals born outside of the Western Pacific, including Canadian-born Aboriginals and Canadian-born non-Aboriginals.

† Independent of sex and age at diagnosis; non-Beijing strains are the reference group.

‡ Of respiratory cases, 440 cases among those born in the Western Pacific and 860 cases among those born elsewhere had smear microscopy data.

§ p = 0.057.

** p = 0.069.

†† Sputum smear positive specimens were collected for 88 foreign-born Western Pacific and 233 ‘Other’ respiratory TB cases.

‡‡ p = 0.062.

**Table 4:** Association between *M. tuberculosis* lineage and disease presentation based on age at diagnosis.

	<35 years at diagnosis			≥35 years at diagnosis		
	Beijing	Non-Beijing		Beijing	Non-Beijing	
	(n = 104)	(n = 438)		(n = 246)	(n = 1038)	
Disease Presentation	No. (%)	No. (%)	aOR (95% CI)*	No. (%)	No. (%)	aOR (95% CI)*
Respiratory TB	73 (70.2)	319 (72.8)	1.6 (0.9, 2.7)	186 (75.6)	803 (77.4)	1.3 (0.9, 1.9)
Sputum smear-positive†	34 (48.6)	128 (42.5)	1.9 (1.0, 3.4)‡	78 (44.8)	410 (54.3)	0.9 (0.6, 1.4)
High bacillary load§	7 (36.8)	18 (31.6)	8.1 (0.9, 74.6)	9 (25.7)	71 (33.8)	0.9 (0.3, 2.2)
Lung cavitation	23 (22.1)	81 (18.5)	1.7 (1.0, 3.1)∥	29 (11.8)	187 (18.0)	0.7 (0.5, 1.2)
Immediately life-threatening TB	4 (3.8)	15 (3.4)	1.5 (0.4, 5.1)	12 (4.9)	84 (8.1)	0.7 (0.4, 1.5)
Any first-line drug resistance	31 (29.8)	72 (16.4)	1.3 (0.8, 2.3)	42 (17.1)	89 (8.6)	1.0 (0.6, 1.5)
Monoresistance	15 (14.4)	54 (12.3)	0.8 (0.4, 1.6)	26 (10.6)	66 (6.4)	0.8 (0.5, 1.4)
Polyresistance	13 (12.5)	14 (3.2)	3.1 (1.3, 7.5)	12 (4.9)	20 (1.9)	1.1 (0.5, 2.4)
MDR-TB	3 (2.9)	4 (0.9)	2.4 (0.5, 12.2)	4 (1.6)	3 (0.3)	6.1 (0.9, 42.7)

Abbreviations: aOR, adjusted odds ratio, CI, confidence interval; TB, tuberculosis; MDR-TB, multidrug-resistant tuberculosis; –unable to calculate.

* Independent of sex and origin; non-Beijing strains are the reference group.

† Of respiratory cases, 371 cases among those aged <35 years and 929 cases among those aged ≤35 years had smear microscopy data.

‡ p = 0.042.

§ There were 76 respiratory TB cases diagnosed after 1992 that had sputum smear positive specimens collected on or before the date of diagnosis among those born in the Western Pacific and 245 cases among those born elsewhere.

∥ p = 0.072.

## Discussion

Beijing lineage strains, representing 19% of TB cases in the major-immigrant receiving province of Alberta, have a definite presence within Canada. This presence is largely limited to persons born in the Western Pacific and especially its Beijing ‘hotspots’ of China, South Korea, Japan and Vietnam. Individuals born in the ‘hotspots’ accounted for 81% of Beijing isolates, the rate being nine to 417 times that of the other groups. In particular, Beijing strains had a relatively minimal impact on the incidence of TB in the Canadian-born (incidence of 0.05/100,000 person-years). These findings generally follow that of other low TB incidence immigrant-receiving countries [15], [32], [48]–[50] and further support the well-established correlation between *M. tuberculosis* lineage and the host’s country of origin/birth [31], [42], [51].

Notwithstanding the emergence of Beijing lineage strains in immigrant-receiving countries, there is minimal evidence overall from these countries or this study to indicate that Beijing strains significantly and independently influence the presentation of TB apart from drug resistance [31], [32], [48], [50], [52]–[54]. Beijing strains were significantly more likely than non-Beijing strains in the current study to have polyresistance to first-line antituberculosis drugs independent of demographic factors, clustering, a previous history of TB, or HIV status. A type of polyresistance, MDR-TB, was also three times more likely among Beijing isolates in adjusted analyses. The association between Beijing strains and MDR-TB in immigrant-receiving countries [32], [48], [50] is of epidemiologic and clinical significance given that MDR-TB is one of the greatest threats to global TB control. This is exemplified in Germany where the importation of Beijing strains appears responsible for an increasing occurrence of MDR-TB [22]. The United States’ experience with the MDR strain W also highlights the importance of sustaining effective TB control programs in low incidence settings given the potential for the rapid dissemination of drug-resistant strains of *M. tuberculosis* in healthcare settings, correctional facilities and elsewhere, especially among persons at high risk for TB (e.g. HIV infected persons) [2], [55].

The finding in this study of a borderline association between Beijing strains and respiratory TB accords with associations between these strains and pulmonary TB in studies completed in other immigrant-receiving countries [52], [54]. Post hoc analysis found that this similarity persists when site of disease is changed from ‘respiratory’ (aOR 1.3; CI 1.0–1.8; p = 0.053) to ‘pulmonary’ (aOR 1.3; CI 1.0–1.7; p = 0.055). The association of Beijing strains with extrathoracic TB in the United States [53] appears unique compared to other high-income immigrant-receiving countries [15], [31], [54] and presumably results from variability in the distribution of Beijing sublineages or sample size limitations [56].

Although the relatively consistent association between Beijing lineage strains and respiratory/pulmonary TB has potentially important public health consequences, it is equally important that these strains have not been associated with increased infectiousness in terms of lung cavitation, sputum smear positivity or bacillary load in this and similar studies in immigrant-receiving countries [52], [53], [57]. In the current study there was also no association between Beijing strains and increased disease severity in relation to immediately life-threatening forms of TB. Further, genotyping analysis found that the likelihood of DNA fingerprint clustering was significantly less among Beijing cases than non-Beijing cases both overall and when limited to patients born in the Western Pacific. Together, these findings suggest that the commonly hypothesized hypervirulence and increased transmissibility of Beijing strains is largely unfounded and/or of minimal public health consequence in Alberta, and presumably in other immigrant-receiving areas, when effective TB control programs are in place. By extension, it may have been mere coincidence that Beijing strains led to MDR-TB outbreaks in immigrant-receiving countries as MDR variants in other lineages could have been equally successful, all other factors being equal.

Host-pathogen interactions or other population-specific factors plausibly account for the varied disease presentations highlighted in subgroup analyses. Of interest is that Beijing strains were associated with MDR-TB, polyresistant TB and/or sputum smear-positive disease among individuals born in the Western Pacific or those aged less than 35 years at diagnosis given that similar groups were identified as high-yield targets for routine screening for latent TB infection in Canada [58]. It may therefore be judicious to also take phylogeographical lineages of *M. tuberculosis* into consideration when defining targets for systematic screening for latent TB infection.

Aboriginal peoples are a highly vulnerable population for TB in Canada and are a significant source of TB transmission [40], [41], [59]. The minimal incidence of Beijing strains in Aboriginal peoples further substantiates the growing body of evidence that indicates that immigration has a minimal impact on the epidemiology of TB in the Canadian-born population [41], [60], [61].

Nevertheless, immigration is a decisive factor in the prevalence of Beijing strains within immigrant-receiving countries. For example, the smaller proportion of Beijing strains in the city of Montreal, Canada compared to this study (9% and 19%, respectively) correlates to a substantially smaller proportion of immigrants from the Western Pacific (<15% in Montreal and 25–30% in Alberta) [31], [62]. Geographic areas with a considerable proportion of immigrants from countries dominated by Beijing strains will therefore predictably experience an increased emergence of Beijing strains with epidemiologic patterns not unlike the region of origin.

This is one of the most comprehensive epidemiologic studies of Beijing strains in a high-income immigrant-receiving country to date and is the foremost study of its kind in Canada. The inclusion of a measure of pathogen load was a distinguishing feature of this study that provided further insight into the possible association between *M. tuberculosis* lineage and level of infectiousness. The accuracy of strain classification in this study was enhanced through the use of an unambiguous and validated genotyping methodology [31], [42], [43] as well as secondary genotyping on a substantial sample of isolates at external laboratories. The amalgamation of data from the provincial TB registry and Provincial Laboratory also minimized selection and information bias.

Study limitations include the relatively small number of polyresistant and MDR isolates as well as the limited number of Beijing cases with TB-HIV co-infection, the associated estimates having limited precision. Generalizability of the study findings to other immigrant-receiving countries may be limited by differences in immigration patterns, immigration screening practices, and TB control programs. Within Canada, the study findings are anticipated to be especially relevant to the other major immigrant-receiving provinces of Ontario and British Columbia where approximately 20% and 40%, respectively, of immigrants were born in the Western Pacific [38], [62].

In conclusion, there is little evidence apart from an increased risk of polyresistance or multidrug-resistance to indicate that Beijing strains pose any more of a public health threat than other *M. tuberculosis* strains within a low TB incidence immigrant-receiving country with effective TB control practices in place.

## Supporting Information

Table S1 Countries in the Western Pacific Region of the World Health Organization.
(DOCX)

Table S2 Demographic characteristics of included and excluded *M. tuberculosis* cases in Alberta, 1991–2007.
(DOCX)

Table S3 Associations between *M. tuberculosis* lineage and disease presentation among TB patients aged 15–64 years at diagnosis and with known HIV status.
(DOCX)
