# Supplementary material for: A Population-Based Cohort Study of Mycobacterium tuberculosis Beijing Strains: An Emerging Public Health Threat in an Immigrant-Receiving Country?
Source: PLoS One. 2012 Jun 5;7(6):e38431. doi: 10.1371/journal.pone.0038431 (PMC3367965; doi:10.1371/journal.pone.0038431)
Supplement: Table S1 — Countries in the Western Pacific Region of the World Health Organization. (DOCX) [file pone.0038431.s001.docx]

**Table S1.** Countries in the Western Pacific Region of the World Health Organization.

| American Samoa | New Caledonia |
| --- | --- |
| Australia | New Zealand |
| Brunei Darussalam | Niue |
| Cambodia | Northern Mariana Islands |
| China^*^ | Palau |
| Cook Islands | Papua New Guinea |
| Fiji | Philippines |
| French Polynesia | Pitcairn Islands |
| Guam | Samoa |
| Japan | Singapore |
| Kiribati | Solomon Islands |
| Korea, Republic of | Tokelau |
| Lao People's Democratic Republic | Tonga |
| Malaysia | Tuvalu |
| Marshall Islands | Vanuatu |
| Micronesia, Federated States of | Vietnam |
| Mongolia | Wallis and Futuna |
| Nauru |  |

* Includes the Special Administrative Regions of Hong Kong and Macau as well as the Republic of China (Taiwan)
