# Supplementary material for: A Population-Based Cohort Study of Mycobacterium tuberculosis Beijing Strains: An Emerging Public Health Threat in an Immigrant-Receiving Country?
Source: PLoS One. 2012 Jun 5;7(6):e38431. doi: 10.1371/journal.pone.0038431 (PMC3367965; doi:10.1371/journal.pone.0038431)
Supplement: Table S2 — Demographic characteristics of included and excluded M. tuberculosis cases in Alberta, 1991–2007. (DOCX) [file pone.0038431.s002.docx]

**Table S2.** Demographic characteristics of included and excluded *M. tuberculosis* cases in Alberta, 1991-2007.

|  |  | **Included Cases** | **Excluded Cases** | **p-value*** |
| --- | --- | --- | --- | --- |
| **Group** | **Subgroup** | No. (%) | No. (%) |  |
| Sex | Female | 877 (48.0) | 17 (65.4) | 0.077 |
|  | Male | 949 (52.0) | 9 (34.6) |  |
| Age at Diagnosis | <35 years | 541 (29.6) | 6 (23.1) | 0.065 |
|  | 35-64 years | 693 (38.0) | 6 (23.1) |  |
|  | >64 years | 592 (32.4) | 14 (53.9) |  |
| Population Group | Canadian-born Aboriginal | 294 (16.1) | 5 (19.2) | 0.623 |
|  | Canadian-born Other | 339 (18.6) | 7 (26.9) |  |
|  | Foreign-born Western Pacific | 693 (38.0) | 9 (34.6) |  |
|  | Foreign-born Other | 500 (27.4) | 5 (19.2) |  |
| Total |  | 1826 | 26 |  |

*Obtained with univariate logistic regression.
