# Supplementary material for: A Population-Based Cohort Study of Mycobacterium tuberculosis Beijing Strains: An Emerging Public Health Threat in an Immigrant-Receiving Country?
Source: PLoS One. 2012 Jun 5;7(6):e38431. doi: 10.1371/journal.pone.0038431 (PMC3367965; doi:10.1371/journal.pone.0038431)
Supplement: Table S3 — Associations between M. tuberculosis lineage and disease presentation among TB patients aged 15–64 years at diagnosis and with known HIV status. (DOCX) [file pone.0038431.s003.docx]

**Table S3.** Associations between *M. tuberculosis* lineage and disease presentation among TB patients aged 15-64 years at diagnosis and with known HIV status.

|  | **Beijing (n=103)** | **Non-Beijing (n=602)** |  |  |
| --- | --- | --- | --- | --- |
| **Disease Presentation** | **No. (%)** | **No. (%)** | **OR (95% CI)*** | **aOR (95% CI)**† |
| Respiratory TB | 74 (71.8) | 454 (75.4) | 0.8 (0.5, 1.3) | 1.5 (0.9, 2.5) |
| Sputum smear positive‡ | 40 (58.0) | 239 (56.4) | 1.1 (0.6, 1.8) | 1.5 (0.8, 2.6) |
| High bacillary load§ | 11 (36.7) | 57 (37.3) | 1.0 (0.4, 2.2) | 1.4 (0.5, 3.5) |
| Lung cavitation | 19 (18.4) | 137 (22.8) | 0.8 (0.5, 1.3) | 1.0 (0.6, 1.9) |
| Immediately life-threatening TB | 5 (4.9) | 46 (7.6) | 0.6 (0.2, 1.6) | 0.7 (0.2, 1.9) |
| Any first-line drug resistance | 29 (28.2) | 75 (12.5) | 2.8 (1.7, 4.5) | 1.5 (0.9, 2.6) |
| Monoresistance | 13(12.6) | 53 (8.8) | 1.7 (0.9, 3.4) | 1.0 (0.5, 2.0) |
| Polyresistance | 13 (12.6) | 16 (2.7) | 5.8 (2.7, 12.5) | 2.8 (1.2, 6.7) |
| MDR-TB | 3 (2.9) | 6 (1.0) | 3.6 (0.9, 14.5) | 2.1 (0.4, 10.1) |
| TB-HIV co-infection | 5 (4.9) | 46 (7.6) | 0.6 (0.2, 1.6) | 1.4 (0.5, 4.2) |

Abbreviations: TB, tuberculosis; MDR-TB, multidrug-resistant TB; HIV, human immunodeficiency virus; OR, odds ratio; aOR, adjusted odds ratio; CI, confidence interval.

* Non-Beijing lineage strains are the reference group.

† Adjusted for sex, age, population group and HIV status; non-Beijing lineage strains are the reference group.

‡ Sputum smear microscopy was completed on 493 respiratory cases with known HIV status. Of these, 69 (14%) were Beijing lineage strains and 424 (86%) were non-Beijing lineage strains.

§183 respiratory TB cases diagnosed after 1992 had sputum smear positive specimens collected on or before the data of diagnosis. Of these, 30 (16%) were Beijing lineage strains and 153 (84%) were non-Beijing lineage strains.
